# Ultrasensitive and Selective Fluorescent Sensor for 5-Hydroxymethylfurfural Based on a Molecularly Imprinted Polymeric Nanocomposite

**DOI:** 10.3390/polym17202799

**Published:** 2025-10-20

**Authors:** Fatih Pekdemir, İzzet Koçak

**Affiliations:** 1 Department of Chemistry, Faculty of Science, Zonguldak Bülent Ecevit University, Farabi Campus, 67800 Zonguldak, Turkey; 2 Division of Pharmaceutical Basic Sciences, Faculty of Pharmacy, Zonguldak Bülent Ecevit University, İbn-I Sina Campus, 67800 Zonguldak, Turkey; izzetkocak@beun.edu.tr

**Keywords:** molecularly imprinted polymer, MOF, N-GQD, nanocomposite, HMF

## Abstract

A fluorescence sensor was designed based on nitrogen-doped graphene quantum dots confined in a metal–organic framework and molecularly imprinted polymer for the selective determination of 5-hydroxymethylfurfural (HMF). Morphological, structural, and spectroscopic characterizations, such as SEM, STEM, BET, FT-IR, and XRD, verified successful synthesis and imprinting with enhanced surface area and structural durability. The sensor demonstrated intense fluorescence at around 420 nm, which was quenched through photoinduced electron transfer (PET) by HMF, exhibiting a linear relationship up to 35 µmol L^−1^ and a detection limit of 30 nmol L^−1^. It offered high imprinting efficiency, selectivity, and stability. The sensing platform also displayed efficient anti-interference performance toward interference species and presented excellent recovery in actual food samples such as honey, juice, and coffee, thus revealing the applicability of the sensing device for real-world HMF measurement in complicated matrices.

## 1. Introduction

The thermal dehydration of hexose sugars, such as glucose and fructose, is the primary reaction that yields 5-hydroxymethylfurfural (HMF) [1,2,3]. This phenomenon is typically derived from the application of heat-based food manufacturing procedures, including baking, roasting, drying, and caramelisation [4]. Therefore, HMF is a prevalent compound in a broad range of foodstuffs, notably those with a relatively high sugar content, including honey, fruit juices, coffee, bakery foods, and processed milk [5]. The existence of HMF in edibles has been a cause for concern, owing to its possible effects on public health. Although HMF is not considered to be acutely or chronically poisonous at nutritional doses, its metabolic conversion into 5-sulfooxymethylfurfural (SMF)—a reactive intermediate—has been demonstrated to be genotoxic and mutagenic in vivo studies [6,7]. Numerous in vitro and in vivo experiments have indicated that long-term exposure to HMF may result in a range of adverse effects, including oxidative stress, inflammatory responses, and DNA damage, which may lead to serious long-term diseases, such as cancer and neurodegenerative diseases [8,9]. Therefore, HMF is gradually becoming an established marker of food safety, with its existence subject to strict regulation in several countries, notably in the areas of honey and fruit-based dietary products [10,11,12]. In view of these potential health hazards, the determination of HMF content in food products has emerged as a significant component of quality control and regulatory compliance.

A variety of analytical techniques have been employed for the identification of HMF in complicated food substances, such as UV-Vis spectrophotometry, HPLC, GC, and CE. Each offering its strengths and weaknesses in terms of selectivity and sensitivity [11,13,14]. The aforementioned instrumental methods are not devoid of drawbacks; for instance, the costs of required equipment and complicated operational conditions [15,16]. In recent times, fluorescence spectroscopy has been identified as a probable replacement for HMF determination on account of its intrinsic benefits. The utilisation of this method with nanoparticles such as quantum or carbon dots is predicated on its high sensitivity and low detection limits, which render it a viable choice for trace analysis [17]. In comparison with conventional chromatography, fluorescence spectroscopy is a less time-consuming, more cost-effective, efficient, and eco-friendly strategy, especially when employed in combination with compact or minimised devices [18].

Graphene quantum dots (GQDs), a novel generation of zero-dimensional carbon nanomaterials, have recently emerged as a major focus in research due to their enhanced physicochemical features [19]. The nanostructures with diameters typically less than 10 nm possess intrinsic photoluminescence, high surface area-to-volume ratios, good aqueous dispersibility, and excellent chemical stability [20]. When integrated into fluorescence sensing platforms, GQDs serve as versatile and stable fluorescent materials, as well as highly efficient signal modulators, a consequence of their substantial quantum confinement and edge effects [21]. The excitation-tunable and size-dependent fluorescence characteristics of these molecules render them particularly suitable for the construction of highly sensitive and specific optical sensors [22]. It has been demonstrated that the most effective method of enhancing the efficacy of GQD-based sensors is through the implementation of heteroatom doping with N, B, P, and S [23]. The integration of these dopants results in the formation of local electronic states, thereby modulating the band structure of GQDs and consequently enhancing their optical, electronic, and chemical characteristics [24]. Nitrogen doping, for instance, has been shown to have a significant impact on the electron density, with the addition of electron pairs to the carbon backbone resulting in enhanced photoluminescence quantum yield, resulting in brighter fluorescence emission and higher stability towards in vivo and environmental circumstances [25,26].

The combination of N-doped graphene quantum dot (N-GQD) derived fluorescent sensors with metal–organic frameworks (MOFs) may offer lots of synergistic properties for the specific and accurate detection of 5-hydroxymethylfurfural (HMF). MOFs, crystalline porous structures composed of metal ions or clusters linked with organic ligands, are known to possess a high surface area, tunable porosity, and versatile, customisable features [27]. The structural characteristics under consideration permit the efficacious preconcentration of analyte molecules by means of both physical adsorption and host–guest mechanism, thus resulting in the enhancement of the local concentration of the analyte in the vicinity of the detection component [27]. Incorporating GQDs into the MOF matrix has been demonstrated to enhance the dispersion and durability of the quantum dots, while concurrently functioning as a defensive barrier that maintains their fluorescent characteristics, regardless of the surrounding environment [28]. In addition, the porosity of MOF provides a pathway for efficient mass transfer and diffusion of the target molecule via the fluorescent nanosystem, thereby enhancing signal and expediting the interactivity between the analyte and the fluorescent nanocomposite [29,30].

The implementation of a molecularly imprinted polymer (MIP) layer within the N-GQD-MOF composite structure can be identified as an enhancement in molecular recognition capacity for the fluorescence probe because the polymer coating around N-GQD-MOF might be expected to contribute to the sensor’s selectivity via the generation of site-specific binding structures that exhibit both spatial and chemical affinity with the HMF molecule. These unique imprinted regions have been shown to facilitate selective rebinding of analyte, despite the co-existence of structurally related interfering substances, which underlie the capacity for precise recognition within complex food matrices [31,32]. The employment of the functional monomer facilitates the occurrence of π–π interactions and hydrogen bonding with the analyte, consequently rendering the affinity and selectivity of the binding sites [33,34,35]. The characteristic of the polymeric coating is its function as a molecular sieve, which serves to minimise nonspecific adsorption and background noise, hence resulting in an enhancement of the signal-to-noise ratio and an improvement in detection sensitivity [36]. Furthermore, the near-neighbourhood of the imprinted binding regions to the fluorescent centre facilitates the conversion of interaction into quantifiable fluorescence signals, by means of mechanisms including photoinduced electron transfer (PET), fluorescence quenching, and Förster resonance energy transfer (FRET) [37,38,39].

To overcome the inherent drawbacks of the reported fluorescent HMF sensors, our strategy integrates nitrogen-doped graphene quantum dots (N-GQDs), a porous metal–organic framework (MOF), and a thiophene acetic acid (TAA)-derived molecularly imprinted polymer (MIP) as a single sensing platform. Every component of the hybrid has a synergistic role: N-GQDs have high photoluminescence but typically experience aggregation-induced quenching and interference of the background; encapsulation within a crystalline MOF matrix helps disperse them, protects the fluorescent core, and simultaneously enhances the local concentration of analytes since MOFs are highly porous and have tunable adsorption capacity. The subsequent coating of a TAA-based MIP layer adds strongly selective recognition cavities that reduce non-specific quenching, improve the imprinting factor, and improve overall selectivity and stability. Such tri-component integration thus counters three of the most common shortfalls of earlier HMF sensors—quenching interference, poor imprinting efficiency, and limited long-term stability. Compared to the most sensitive [6,7,8,9,10,11,12,13,14,15,16,17,18,19,20,21,22,23,24,25,26,27,28,29,30,31,32,33,34,35,36,37,38,39,40] reported to date (Table S1), ratiometric fluorescent probes [40] and graphitic carbon nitrite-based nanosensors [6], the present architecture provides a comparable detection limit and imprinting factor and much improved selectivity, stability, and reproducibility, all combined, making it a unique and superior sensing structure. In consideration of the aforementioned points, it is evident that the N-GQDs offer a fluorescent, robust core, whilst the MOF configuration provides an accessible, chemically tunable platform for dispersion and analyte accessibility. The surface imprinting results in the selective incorporation of molecular recognition, thereby enabling the sensor to identify HMF in the presence of structural analogues. The utilisation of such a structural arrangement within the sensor device was also demonstrated to enhance the fluorescence signal through a synergistic effect, while ensuring high selectivity and chemical stability. In addition, the study is also a tendency towards employing multifunctional and biologically synthesized nanomaterials for sensing devices. For example, Razzaque et al. [41] have recently combined Kraft lignin with graphitic carbon nitride and nickel for achieving ultrasensitive electrochemical pesticide sensing. Therefore, it would be fairly reasonable to conclude that our approach is in accordance with recent advances in hybrid nanocomposite sensors, while also extending the notion to fluorescence-based sensing and food safety analysis.

To the best of our knowledge, this represents the first example of a fluorescence sensor that incorporates N-GQD, MOF, and thiopheneacetic acid (TAA)-based imprinted polymer for the particular sensing of HMF. The novel architecture presented herein opens up new opportunities for the fabrication of highly selective, efficient, and cost-effective sensors that have the potential to be applied in food safety analysis and quality control, and also offers a structurally rational and performance-driven solution to the limitations of prior systems.

## 2. Materials and Methods

Na_2_HPO_4_, NaH_2_PO_4_, NaCl, ascorbic acid (AA), Tris, HMF, 5-methyl furfural, acetone, and caffeine were purchased from Merck (Darmstadt, Germany). Azobisisobutyronitrile (AIBN), chelidamic acid, Ethylene glycol dimethacrylate (EGDMA), Glucose, fructose, maltose, sucrose, dopamine, lactose, NaOH, L-cysteine, citric acid, glutamic acid, acetonitrile, and metal chlorides were also obtained from Sigma Aldrich (St. Louis, MO, USA). Ethanol, methanol, glycine, cysteine, gallic acid anduric acid, were also purchased from Fisher Scientific (Pittsburgh, PA, USA). Thiophene acetic acid (TAA) was also obtained from Chem Bio (İstanbul, Turkey).

All fluorescence measurements of the composite materials were carried out using an PerkinElmer LS-55 fluorescence spectrometer (Shelton, CT, USA). Initially, full-spectrum scans were conducted to determine the optimal excitation wavelength for assessing the fluorescence characteristics of the composites. Based on these scans, an excitation wavelength of 280 nm was selected, and emission spectra were recorded over the 200–600 nm range. The working solutions were prepared by dispersing a defined amount of N-GQD-MOF-MIP or N-GQD-MOF-NIP in phosphate-buffered saline (PBS), followed by ultrasonication to ensure uniform dispersion, resulting in a stock solution with a concentration of 1 mg/3 mL. Various concentrations of HMF were then introduced into the fluorescent cuvettes, and the corresponding fluorescence intensities were measured.

The morphological characteristics of N-GQD-MOF and N-GQD-MOF-MIP were investigated using SEM and STEM techniques with the aid of TESCAN MAIA3 XMU equipment (Brno, Czech Republic). Elemental analysis was carried out by energy-dispersive X-ray spectroscopy (EDX). Crystallographic structures were examined via X-ray diffraction (XRD) using the Rigaku Smart Lab (Tokyo, Japan). Nitrogen adsorption–desorption isotherms were measured with an ASAP 2020 surface area analyzer from Micromeritics (Norcross, GA, USA) to assess the specific surface area and porosity. Fourier transform infrared (FT-IR) spectra in the range of 4000–500 cm^−1^ were recorded using KBr pellets on a PerkinElmer FTIR Spectra 100 (Shelton, CT, USA). UV–Vis absorption spectra were obtained using a Varian Carry 100 Bio spectrophotometer (Palo Alto, CA, USA).

For the quantitative evaluation of real samples, samples of commercially purchased from local store, coffee, honey, and fruit juices underwent to pre-treatment procedure before fluorometric measurements. A known amount of each sample was accurately weighed and dissolved in deionized water for the purposes of gaining adequate solubility and maintaining the correct analyte concentration. The resulting suspensions were sonicated for 30 min to achieve thorough dissolution and homogenization, and centrifuged at 5000 rpm for 30 min to decant suspended particulates and insoluble residues. The supernatant was subsequently decanted carefully and filtered through a 0.45 µm syringe filter to eliminate any remaining fine particles.

### 2.1. Synthesis of N-GQD

The synthesis of N-GQD was carried out by slightly modifying protocols described in earlier studies [42,43]. In a typical procedure, 2.0 g of citric acid (10.4 mmol) and 1.0 g of Tris(hydroxymethyl)aminomethane (8.25 mmol) were dissolved in 20 mL of ultrapure water. The resulting solution was placed into a Teflon-lined stainless steel autoclave and heated at 200 °C for 4 h. After the mixture was allowed to cool down to room temperature, a bright orange color was observed, indicating the formation of N-GQD. The solution was then carefully neutralized with 1.0 M NaOH until the pH reached 7.0. To eliminate any remaining particulates or impurities, it was filtered through a 0.22 µm PTFE membrane. The resulting N-GQD dispersion was stored at 4 °C and used directly without further purification.

### 2.2. Synthesis N-GQD-MOF

The material was synthesized following a “bottle-around-ship” strategy in accordance with procedures reported in the literature [44,45]. ZnSO_4_·7H_2_O (125 mg, 0.43 mmol) was dissolved in 10 mL of an N-GQD solution, while chelidamic acid (85 mg, 0.46 mmol) was dissolved in 2 mL of ethanol. The two solutions were mixed together and poured into a Teflon-lined stainless steel autoclave. The sealed container was then heated at 180 °C for three days. After that, it was simply left to cool down on its own to room temperature. The resulting dark reddish-brown liquid was concentrated by removing the solvent under vacuum. The leftover solid was washed thoroughly a couple of times with hot methanol to get rid of any impurities. Finally, the product was dried in a vacuum oven at 60 °C for two days, yielding about 100 mg of a brown solid.

### 2.3. Synthesis of N-GQD-MOF-MIP

**Scheme 1:**
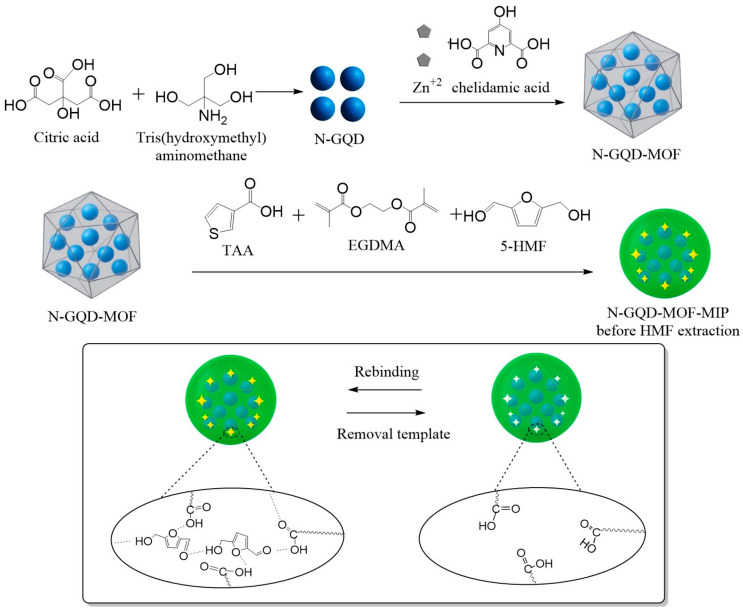
Systematic representation for the development of N-GQD-MOF-MIP-based fluorescence sensor.

MIP synthesis on the obtained N-GQD-MOF-MIP was achieved in accordance with existing literature, with slight variations [46,47]. In a 25 mL round-bottom flask, 35 mg of N-GQD-MOF was dispersed by stirring in 4 mL of DMF. Subsequently, 10 mg of 5-(Hydroxymethyl)furfural (HMF) and 30 µL of 3-thiopheneacetic acid (TAA) were added, and the mixture was stirred at room temperature for 30 min. Afterwards, 200 µL of ethylene glycol dimethacrylate (EGDMA) and 20 mg of 2,2′-azobis(2-methylpropionitrile) (AIBN) were introduced. The flask was then sealed under a nitrogen atmosphere and stirred in a water bath at 60 °C for 3 h. At the end of the reaction, a gel-like pale green solid was obtained. This product was washed thoroughly with excess ethyl ether and dried in a desiccator. A total of 150 mg of resulting N-GQD-MOF-MIP solid was transferred into a Soxhlet extractor and subjected to extraction using a methanol: acetic acid mixture (9:1, 200 mL) through five siphon cycles. Following the extraction, the solid residue was thoroughly rinsed with methanol and ethyl ether, then stored in a desiccator until further use. The N-GQD-MOF-NIP synthesis was performed using the same protocol as the MIP synthesis, with the exception that HMF was not used. In this instance, the final solid showed a lighter greenish hue. The details of all the steps involved in the production of the fluorescence sensor was also given in Scheme 1.

## 3. Results and Discussion

**Figure 1:**
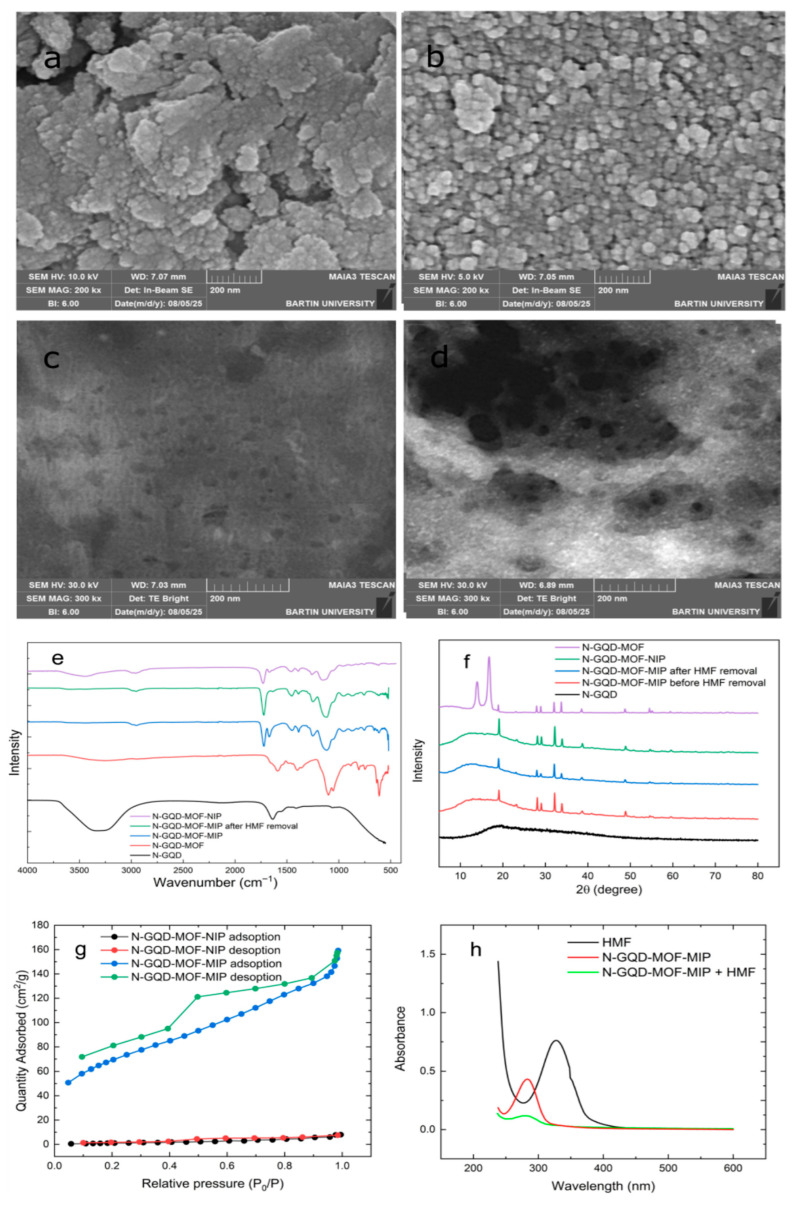
SEM images and STEM images of N-GQD-MOF (**a**,**c**), N-GQD-MOF-MIP (**b**,**d**). FTIR spectra (**e**) and X-ray diffraction patterns (**f**) of N-GQD, N-GQD-MOF, N-GQD-MOF-MIP before and after template removal and N-GQD-MOF-NIP. Nitrogen adsorption and desorption curves (**g**) of N-GQD-MOF-MIP and N-GQD-MOF-NIP. UV spectra (**h**) of N-GQD, N-GQD-MOF-MIP, and N-GQD-MOF-MIP + HMF.

The surface morphology of N-GQD-MOF and N-GQD-MOF-MIP was examined in detail by the SEM technique, and the images obtained are shown in Figure 1a,b. It is evident that after MIP coating, the surface underwent a complete transformation, exhibiting increased roughness in specific areas, and the edges of individual particles became less distinguishable. This type of coating is observed to result in the blurring of particle outlines, a phenomenon that may underlie the more merged and less well-defined texture evident in the images. This result is certainly ascribed to the successful generation of polymeric layers over the solid framework of N-GQD-MOF, referring to the fact that the MIP layer around the structure is effectively formed, thus enabling the creation of molecular recognition centers at the surface. EDX analysis is also performed to provide additional information regarding the composition of both relevant materials. In the case of N-GQD-MOF, carbon, oxygen, and zinc are found to be present, which is in accordance with the anticipated architecture of the framework. However, in the analysis of N-GQD-MOF-MIP, sulfur was identified in the obtained spectrum, most probably as a consequence of the thiophene monomer, alongside a significant enhancement in oxygen content. It can be concluded from these outcomes that the MIP layer was effectively incorporated, and the sustained presence of Zn is indicative of the integrity of the MOF. The results of the EDX analysis are also provided in Figures S1 and S2.

As displayed in Figure 1c,d, STEM imaging was also carried out to determine the purpose of determining dimensions and the structural features of fabricated both nanocomposite materials. The N-GQD-MOF displayed a uniform and even distribution of particles, with an average diameter of approximately 16–23 nm. This observation is indicative of the effective dispersion of the N-GQD within the MOF. Following the imprinting procedure, the N-GQD-MOF-MIP sample exhibited enhanced contrast and particle dimension differences. The size of the particles exhibited a greater variation, which can be associated with the formation of a polymeric shell over the N-GQD-MOF.

To reveal the successful chemical conversions in each synthesised process, FT-IR spectra of N-GQD, N-GQD-MOF, and N-GQD-MOF-MIP before and after the removal of the template were presented in Figure 1e. The broad peak at 3324 cm^−1^ in the spectrum may be attributed to O–H and N–H group stretching vibrations, indicating abundant surface hydroxyl and amino functionalities that enhance hydrophilicity and provide reactive sites for subsequent modification steps. Peaks at 1635 and 1556 cm^−1^ could be attributable to C=O (carbonyl) and C=C vibrations in aromatic rings, respectively, suggesting the presence of conjugated π-systems that contribute to their fluorescence properties. Peak at 1410 cm^−1^ is assigned to C–N stretching vibrations, while the peak at 1059 cm^−1^ corresponds to the C–OH vibrations of hydroxyl groups for the N-GQD [43,48]. Additionally, the broad peak at 3260 cm^−1^ is indicative of –OH groups, and the peak at 2933 cm^−1^ can also correspond to C–H stretching vibrations. Peaks at 1590 and 1054 cm^−1^ correspond to C=N and C–N stretching vibrations of the pyridine ring (the pyridine ring is referring to the chelidamic acid moiety in the MOF), respectively, and also a shoulder at about 1590 cm^−1^ and the 1357 cm^−1^ are responsible for asymmetric and symmetric stretching vibrations of carboxylate groups (COO^−^) coordinated to the benzene ring [44,49]. The peak at the 1399 cm^−1^ can be associated with C–H bending vibrations, while the peak at about 1100 cm^−1^ corresponds to C–O–C absorption by carboxyl esters [50]. The absorption peaks at 747, 611, and 529 cm^−1^ are indicative of some Zn–O and Zn–N bonds, which suggest the successful formation of the N-GQD-MOF and further supports the establishment of a stable metal–ligand framework in which N-GQDs are successfully embedded within the MOF matrix [51,52]. On polymerization of the N-GQD-MOF structure in the presence of the template molecule of HMF, the band located at 1721 cm^−1^ in the spectrum is attributed to the stretching vibrations of carboxyl groups of the polymerization reagents of TAA and EGDMA, while the band present at 1670 cm^−1^ is assigned to the C=O stretching vibration of the conjugated aldehyde group of HMF, which is in good agreement with the N-GQD-MOF-MIP [47,53]. Overall, these observations are a sign of successful integration of the target molecule HMF into the structure. Following Soxhlet extraction of N-GQD-MOF-MIP, disappearance of aldehyde carbonyl (C=O) stretching vibration band at 1670 cm^−1^ in the FT-IR spectrum testifies to successful extraction of the HMF molecule from the material, which implies the formation of complementary cavities capable of recognizing HMF via hydrogen bonding and π–π interactions with the remaining functional groups (–OH, –NH, and C=O).

As depicted in Figure 1f, the crystalline structure of each material synthesised during the sensing platform acquisition process was examined using the XRD technique. Upon initial analysis of the XRD pattern for the N-GQD-MOF, a series of notably sharp and well-defined peaks, situated between 10° and 40°, were detected, which is frequently linked to the high degree of crystallinity in the material under investigation [51]. However, N-GQD typically results in the formation of a broad peak around 19° [54,55], but no significant disruption to the crystallinity of the MOF was observed [56]. A subtle increase in background signal was recorded, which is likely attributable to the inherently amorphous and graphitic properties of N-GQDs [57,58]. Following the incorporation of the polymeric layer to form MIP, the diffraction peaks from the MOF became absent, particularly between 10° and 35°. It can be hypothesised that the disappearance of the corresponding peak likely stems from the polymer coating, which introduces a degree of disorder that causes observed spectral changes [50]. However, the absence of additional peaks suggests that the polymer is amorphous. Subsequent to the removal of the template molecule, the XRD pattern remained virtually identical, thereby indicating that the MOF structure retained its integrity throughout the removal procedure. The N-GQD-MOF-NIP demonstrated a remarkably similar pattern to the MIP. MOF peaks remained present, but their intensity was reduced. Given that the decrease in intensity was similar in both cases, we can infer that it is due to the polymer coating, rather than any structural damage to the MOF, which led to the observed change [59].

The nitrogen adsorption/desorption isotherms at 77 K were utilised to examine the surface area and porosity of the imprinted and non-imprinted polymeric structures, as displayed in Figure 1g. The Brunauer–Emmett–Teller (BET) surface area of N-GQD-MOF-MIP and N-GQD-MOF-NIP was determined to be 249 m^2^ g^−1^ and 5.5 m^2^ g^−1^, respectively. It is evident that the surface area of the imprinted polymer (MIP)-based composite is considerably higher, thereby validating the efficacy of the imprinting process, which successfully resulted in the creation of specific recognition cavities within the polymer matrix, generated as a consequence of the extraction of the template molecule. N-GQD-MOF-NIP, conversely, is deficient in a structural characteristic and thus possesses a significantly decreased surface area.

It is well-known that the majority of sensor platforms are operated with respect to the principles of fluorescence quenching, predominantly through either dynamic or static mechanisms. During the process of static quenching, a fluorescent molecule and a target molecule can form a complex in the ground state, thus preventing the emission of light. Such interactions are known to induce alterations in the UV-vis absorption spectra [60]. In our study, we compared the UV–vis spectra of HMF, N-GQD-MOF-MIP, and N-GQD-MOF-MIP + HMF, as shown in Figure 1h. The lack of any new or distinct absorption peaks suggests that no ground stable complex was formed and that there was no meaningful chemical interaction between N-GQD-MOF-MIP and HMF. Static quenching frequently results in significant changes to the UV-vis spectrum, owing to the formation of a ground-state complex. However, the absence of such changes implies that static quenching does not play a role in the sensing mechanism [61,62].

**Figure 2:**
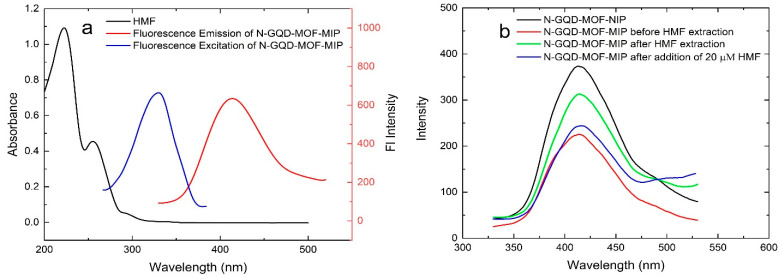
UV–vis absorption spectra of HMF, Fluorescence excitation and emission spectra of the N-GQD-MOF-MIP (**a**). Fluorescence spectra of N-GQD-MOF-NIP, N-GQD-MOF-MIP before and after removal of template, and template-free N-GQD-MOF-MIP after addition of 20 μM of HMF (**b**).

Additionally, as demonstrated in Figure 2a, it is evident that the fluorescence emission of N-GQD-MOF-MIP does not align with the absorption spectrum of HMF. This effectively rules out any possibility of the inner filter effect or Förster resonance energy transfer [62]. It can thus be concluded that the quenching that occurs in this instance is more likely to be a result of photoinduced electron transfer (PET) [59]. It is also noteworthy that the conduction band of the N-GQD-MOF-MIP appears to demonstrate a close alignment with the UV absorption energy level of CPF. Consequently, when the N-GQDs are excited, electrons can move directly into the HMF’s lowest unoccupied molecular orbital. Afterwards, a reversion to the ground state is observed, which is responsible for the decrease in fluorescence intensity. As the HMF concentration increases, hydrogen bonding between HMF and the N-GQD-MOF-MIP surface cavities tends to show enhancement, which thus leads to an increase in the intensity of PET. On the basis of all these explanations, it can be deduced that the greater the strength of the interaction means to the more noticeable the quenching effect on the fluorescence signal of the N-GQD [59]. Figure 2b shows that the N-GQD-MOF-MIP exhibited a fluorescence peak near 420 nm when excited with 280 nm. After removing the template, there was a clear increase in fluorescence—likely because the active binding sites were no longer obstructed. Following the introduction of HMF molecules, a clear decline in the fluorescence intensity was observed, which most probably stems from interactions between HMF and the –COOH groups on the surface of the sensing device, likely facilitated through hydrogen bonding. It appears that when HMF occupies the imprinted cavities, an energy transfer occurs from the excited N-GQD-MOF-MIP to the HMF molecules, which results in a significant drop in the fluorescence signal. Taken together, these results point to the efficacy of N-GQD-MOF-MIP as a detection system for HMF.

**Figure 3:**
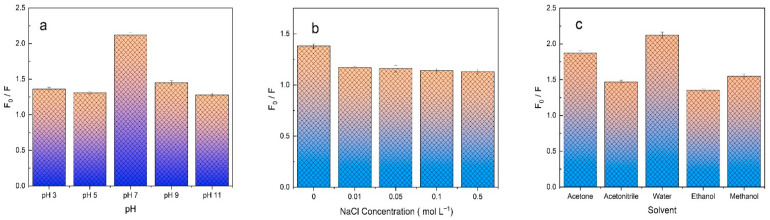
Fluorescence intensity of the N-GQD-MOF-MIP recorded for the investigation of the effect of pH (**a**), effect of ionic strength (**b**), and effect of solvent (**c**).

In order to ensure optimal detection conditions for HMF, it is essential to carefully adjust the pH level, ionic strength, and solvent to disperse the N-GQD-MOF-MIP. It is therefore crucial to note that these factors possess the capability to influence the sensors. Among these factors, pH is considered to be of significant concern due to its ability to impact both the charge state of the HMF and the binding process of the sensing device to HMF. Figure 3a demonstrates that the sensor’s strongest response was obtained at a pH of 7.0, which was consequently identified as the ideal pH value. At more acidic pH values, the number of hydrogen bonds established between the carboxylic groups located on the sensing platform and the template seems to decrease, thereby resulting in lower sensor performance. However, an increase in pH results in the creation of minor surface defects, which can adversely affect the sensor’s sensitivity [58]. As shown in Figure 3b, an investigation was carried out in order to assess the change in the fluorescence intensity of the N-GQD-MOF-MIP sensor when exposed to varying conditions of ionic strength. It is noteworthy that as the concentration of sodium chloride (NaCl) increases, there is only a marginal change in the fluorescence signal, indicating that the sensor retains its efficacy and stability across a wide range of ionic environments. As presented in Figure 3c, dispersing N-GQD-MOF-MIP in water led to a more intense response than in other solvents.

**Figure 4:**
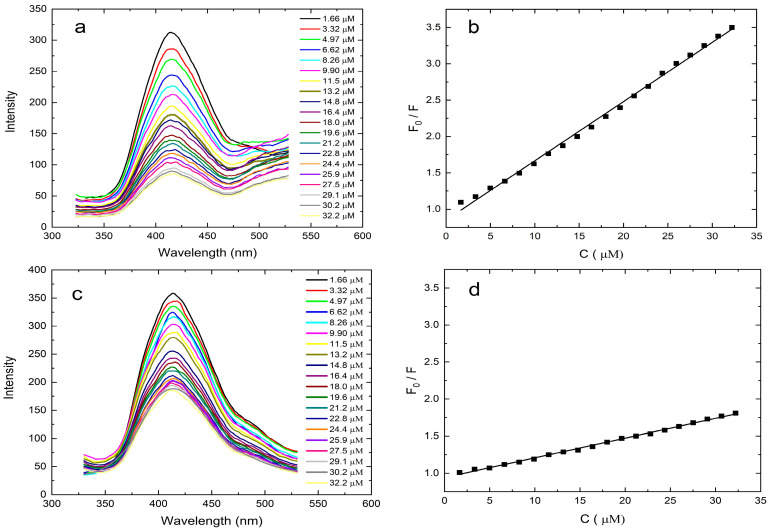
Fluorescence emission spectra of N-GQD-MOF-MIP (**a**) and N-GQD-MOF-NIP (**c**) in the presence of different concentrations of HMF (from 0 to 35 μM). Calibration plots for HMF detection by N-GQD-MOF-MIP (**b**) and N-GQD-MOF-NIP (**d**).

By the addition of different amounts of HMF content ranging from 0 to 35 μM, fluorescence intensities were recorded in order to reveal the fluorescence quenching efficiency and sensitivity of the N-GQD-MOF-MIP and N-GQD-MOF-NIP. As shown in Figure 4a, the emission intensity of N-GQD-MOF-MIP and N-GQD-MOF-NIP decreases gradually with increasing the concentration of HMF, which is caused predominantly by the occupation of unique etched cavities inside N-GQD-MOF-MIP by target molecules. Furthermore, as can be seen in Figure 4c, in spite of the absence of any cavities on the surface, an increment in the added HMF concentration also brings about fluorescence quenching on the N-GQD-MOF-NIP sensing platform, which can be attributed to the adsorption of template molecules. In consideration of the data obtained here, another possible reason for the fluorescence quenching observed on the N-GQD-MOF-MIP surface, which may be negligible, is the non-specific adsorption of target molecules. The relationship between fluorescence intensity and concentration of the template in this system can be described by the Stern-Volmer equation, where *F*_0_ is the fluorescence intensity prior to addition of the template, *F* is the fluorescence intensity upon addition of the template, *K_SV_* is the Stern-Volmer quenching constant, and *C_HMF_* is the concentration of HMF. The calibration curve (Figure 4b,d) from the Stern–Volmer equation indicates that the N-GQD-MOF-MIP and N-GQD-MOF-NIP exhibit a linear response to HMF in the concentration range of 0–35 μM.

(1) $$\frac{F_{0}}{F}=1+K_{SV}[C_{HMF}]$$

The regression equation of N-GQD-MOF-MIP and N-GQD-MOF-NIP are found to be ***F*_0_**/***F*** = 0.9841 + 0.0735[***C_HMF_***] and ***F*_0_**/***F*** = 0.9639 + 0.0243[***C_HMF_***] with the correlation coefficients (R^2^) of 0.9985 and 0.9787, respectively. Meanwhile, the LOD is determined to be 30 nM for N-GQD-MOF-MIP and 1.7 μM for N-GQD-MOF-NIP. Since the LOD of N-GQD-MOF-NIP is calculated to be much greater than N-GQD-MOF-MIPs, it can be inferred that the N-GQD-MOF-MIPs are responsive to HMF and can be utilized as a practical method for the determination of HMF. The results suggest that the performance of the N-GQD-MOF-MIP fluorescence sensor is demonstrated to be superior when compared to other HMF sensors that are documented in the extant literature, as given in Table S1. To evaluate the molecular recognition of the MIP-based sensing platform, the imprinting factor (IF) was calculated as the ratio of the Stern–Volmer quenching constants (***K*_SV_**) for the MIP and NIP. The resulting IF value of 3.06 indicates a significantly enhanced quenching efficiency, suggesting improved spectral recognition of HMF by the developed MIP sensor. These findings demonstrate that N-GQD-MOF-MIP exhibits superior selectivity toward HMF compared to the non-imprinted counterpart.

**Figure 5:**
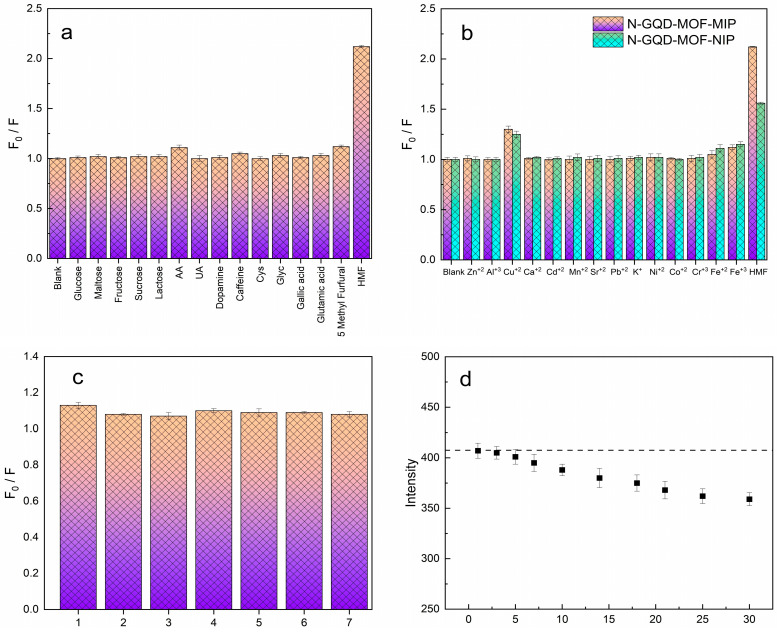
Fluorescence responses of N-GQD-MOF-MIP in the existence of HMF or interfering agents (**a**) Fluorescence responses of N-GQD-MOF-MIP and N-GQD-MOF-NIP in the existence of HMF or metal ions (**b**). Reproducibility of N-GQD-MOF-MIP fluorescent probes for the determination of HMF (**c**). The long-term stability of the fluorescent probe (**d**).

To assess the recognition selectivity of N-GQD-MOF-MIP toward HMF, structurally similar compounds (Glucose, maltose, fructose, sucrose, lactose, ascorbic acid (AA), uric acid (UA), dopamine, caffeine, L-cysteine (Cys), Glycine (Glyc), gallic acid, glutamic acid, 5-methyl furfural) and a range of metal ions (including Ni^2+^, Mn^2+^, Sr^+2^, Cr^+3^, Pb^2+^, Zn^2+^, Ca^2+^, Co^2+^, Cd^2+^, Al^3+^, Cu^2+^, K^+^, Fe^2+^, and Fe^3+^), which are 100 times more concentrated compared to HMF, were considered as potential interferents. Figure 5a highlights a significant decrease in fluorescence intensity upon the addition of HMF to allow it to interact with the MIP surface, whereas other competing compounds only brought about negligible changes. This selective fluorescence response is presumably due to the molecular templating procedure, which creates cavities that are highly selective in terms of size, shape, and chemical properties for the target molecule. Moreover, the co-existence of common metal ions—as illustrated in Figure 5b does not give rise to any substantial interference, despite high concentrations being present. On the other hand, N-GQD-MOF-NIP exhibited only negligible changes in the fluorescence intensities, confirming that the quenching behavior of the imprinted sensor is controlled by selective interactions rather than nonspecific binding. Overall, these results emphasise the excellent selectivity and practical applicability of N-GQD-MOF-MIP for HMF detection, particularly in complex sample matrices.

Molecular recognition selectivity of the imprinted sensor was also examined quantitatively by calculating cross-reactivity ratios (CRRs) between all the possible interfering compounds. CRRs were calculated based on the equation:

(2) $$CRR=\frac{{∆F}_{Analogue}\left(F_{0}-F_{Analouge}\right)}{{∆F}_{HMF}\left(F_{0}-F_{HMF}\right)}$$

**Δ*F_HMF_*** = ***F*_0_** − ***F_HMF_*** is the fluorescence quenching response of the imprinted sensor to HMF at a fixed concentration. **Δ*F_Analogue_*** *= **F*****_0_** − ***F_Analogue_*** is the quenching response under identical conditions for the interfering compound.

All tested analogues had quite lower ***CRR*** values than HMF (***CRR*** = 1.00). Of the monosaccharides, glucose, maltose, fructose, sucrose, and lactose gave 0.048, 0.042, 0.006, 0.012, and 0.012 ***CRRs***, respectively, which are obvious indications of minimal carbohydrate-based compounds interference that is typically present in food matrices. Likewise, uric acid (0.006), dopamine (0.042), cysteine (0.018), glycine (0.054), gallic acid (0.042), and glutamic acid (0.036) provided very low ***CRRs***, and thus the chemically very selective nature of the recognition sites is established. Of all the interferents screened, ascorbic acid (***CRR*** = 0.175) and 5-methylfurfural (***CRR*** = 0.193) produced the biggest responses, which agrees with their redox activity and some structural similarity to HMF. However, the response was below 20% of the HMF signal, thereby demonstrating that the molecularly imprinted layer discriminates strongly against closely related or electroactive species. The findings confirm that the N-GQD-MOF-MIP sensor exhibits superior molecular selectivity, and the imprinting-induced recognition is the reason for the major fluorescence quenching for HMF.

In order to test the reproducibility of the designed sensing device, an experimental setup was established to measure the fluorescence intensities of seven different samples against 5 µM HMF, each tested in three parallel replicates. The results are shown in Figure 5c. The results showed reliable measurements with minimal variation, as reflected by a relative standard deviation (RSD) of 1.79%. In addition, the long-term stability of the fluorescence signal was monitored over a 30-day period (Figure 5d). Although a slight reduction was observed in the fluorescence intensity of N-GQD-MOF-MIP, the sensor retained 85.8% of its original signal, confirming its suitability for long-term applications.

To test the practicality and effectiveness of the proposed method with actual food products, three different types were selected: honey, fruit juice, and coffee. Each sample was spiked with a known amount of HMF at a concentration of 3 µM. High-performance liquid chromatography (HPLC) was used at the same time to confirm the results and determine the method’s accuracy. Recovery rates fell between approximately 95.3% and 98.8%, suggesting that the method performs consistently (see Table 1). The results from this analysis generally aligned well with those obtained by HPLC, with no significant differences observed. Considering all findings, these observations indicate that the N-GQD-MOF-MIP probe can be considered a reliable alternative method for detecting HMF in complex food samples.

To put our N-GQD-MOF-MIP sensor into perspective with the most sensitive fluorescence-based HMF sensors [6,40,63] and other methods such as electrochemical and HPLC [1,16,64,65,66] (Table S1), we compared our analytical figures of merit directly with the two best-performing reports. While reference [40] has an LOD of 21 nM and 94–108% recovery in real samples, reference [6] has an LOD of 4.80 nM with 96–107% recoveries. Although these publications show high sensitivity and accurate recoveries, neither [40] nor [6] reports reproducibility data (RSD) nor long-term operational stability. By comparison, our sensor has an LOD of 30 nM—comparable to the best in the literature—and 95.3–98.8% recoveries in close agreement with HPLC. More importantly, our manuscript introduces additional performance features critical for real-world adoption but not present in the referenced research: we measured reproducibility (RSD = 1.79% for seven independently prepared probes) and long-term stability (probe retained 85.8% of initial fluorescence after 30 days). In terms of molecular recognition, our imprinting factor and cross-reactivity experimentation reveal equivalent selectivity performance to that reported in [40] and [6], while our study examined a larger panel of potential interferents (generic sugars, amino acids, polyphenols, and metal ions), offering higher confidence in real-matrix specificity. Finally, the synthetic and operational procedure of our platform—embedding luminescent N-GQDs in a MOF with a following straightforward surface imprinting step with TAA—provides a simple-to-execute, one-pot-compatible procedure with minimal sample preparation.

## 4. Conclusions

This study presents an efficient and stable method of preparing a fluorescence sensor that is capable of selectively quantifying HMF using nitrogen-doped graphene quantum dots metal–organic framework within a molecularly imprinted polymer matrix. The N-GQD-MOF-MIP composite demonstrated structural robustness, well-dispersed morphology, and a remarkably high surface area, thereby creating specific recognition regions for HMF. The sensor was proven to be both selective and sensitive, with an extremely low detection limit and satisfactory resistance to potential interferents such as metal ions and structurally similar compounds. The proposed N-GQD-MOF-MIP sensor is highly suitable for practical analytical applications in various food matrices, with excellent recovery levels consistent with HPLC analysis. Because of its high sensitivity, high resistance to interferences, simplicity of operation, and long-term stability, the platform can be readily optimized for routine quality control of commercial food and beverage products where HMF must be monitored in real time. The straightforward synthesis process—a one-pot MOF embedding and surface imprinting—facilitates reproducible and scale-up synthesis of sensing films or test strips that, without the requirement of sophisticated instrumentation, can be applied on-site. Beyond HMF, the modularity of the sensor format provides a general strategy for imprinting other small organic analytes with a specific focus on aldehydes, phenolic compounds, and food safety/environmental pollutants. In summary, as the manufactured sensing device was found to exhibit excellent analytical performance, the N-GQD-MOF-MIP sensing platform can be regarded as a promising candidate for the rapid, accurate, and repeatable determination of HMF or other organic substances for inspecting food quality and hygiene.

## Figures and Tables

**Table 1:** The measured HMF content in coffee, honey, and fruit juice products was determined using designed Fluorescent sensor and HPLC (n = 3).

		Fluorescent			HPLC		
Sample	Added (µM)	Found (µM)	Recovery (%)	RSD (%)	Found (µM)	Recovery (%)	RSD (%)
Coffee	3.0	2.96 (±0.14)	98.8	4.73	2.89 (±0.04)	96.2	1.44
Honey	3.0	2.91 (±0.10)	96.9	3.46	2.86 (±0.05)	95.3	1.85
Fruit Juice	3.0	2.92 (±0.08)	97.3	2.72	2.90 (±0.05)	96.8	1.73

## Data Availability

The original contributions presented in this study are included in the article/Supplementary Material. Further inquiries can be directed to the corresponding author.

## Supplementary Materials

The following supporting information can be downloaded at: https://www.mdpi.com/article/10.3390/polym17202799/s1, Figure S1: EDX analysis of N-GQD-MOF; Figure S2: EDX analysis of N-GQD-MOF-MIP; Table S1: The performance of various types of HMF sensors in the literature.
