# Supplementary material for: Ultrasensitive and Selective Fluorescent Sensor for 5-Hydroxymethylfurfural Based on a Molecularly Imprinted Polymeric Nanocomposite
Source: Polymers (Basel). 2025 Oct 20;17(20):2799. doi: 10.3390/polym17202799 (PMC12567061; doi:10.3390/polym17202799)
Supplement: Supplementary file 1 [file polymers-17-02799-s001.zip › polymers-3896987-supplementary.pdf]

polymers

Ultrasensitive and Selective Fluorescent Sensor for 5-Hydroxymethylfurfural Based on a Molecularly Imprinted Polymeric Nanocomposite

Fatih Pekdemir [1,*] and İzzet Koçak [2]

[1] Department of Chemistry, Faculty of Science, Zonguldak Bülent Ecevit University, Farabi Campus, 67800 Zonguldak, Turkey
[2] Division of Pharmaceutical Basic Sciences, Faculty of Pharmacy, Zonguldak Bülent Ecevit University, İbn-I Sina Campus, 67800 Zonguldak, Turkey; izzetkocak@beun.edu.tr
* Correspondence: fatihpekdemir@beun.edu.tr; Tel.: +90-372-257-40-10:

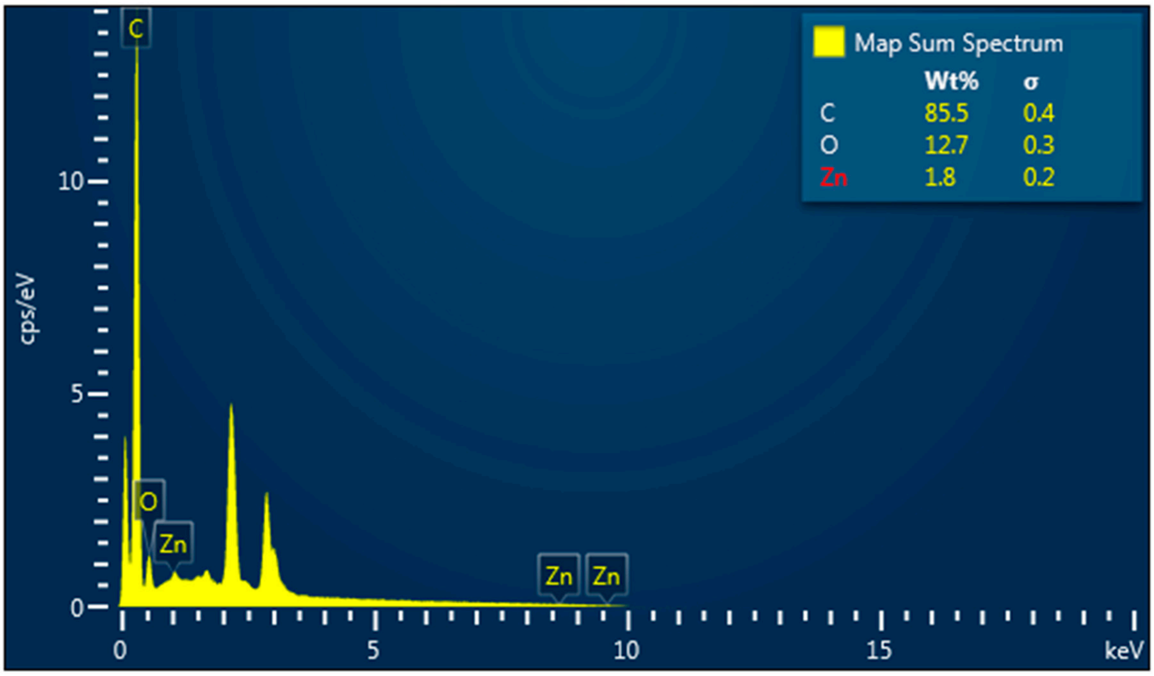

Figure S1. EDX analysis of N-GQD-MOF.

 https://doi.org/10.3390/xxxxx

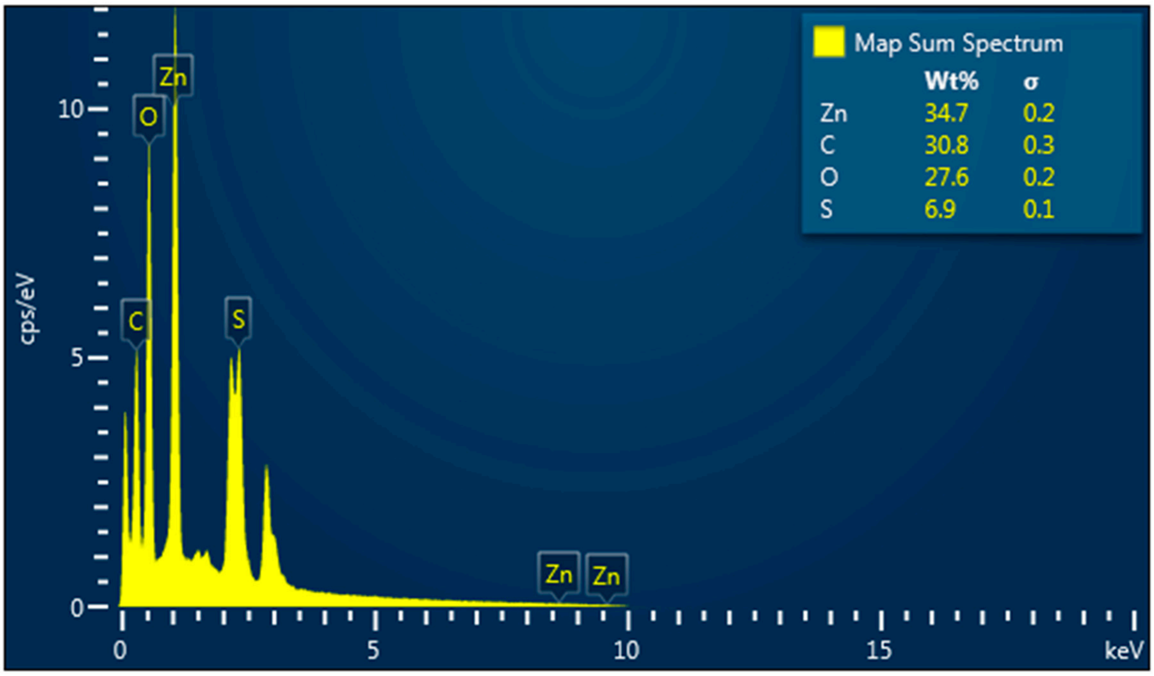

Figure S2. EDX analysis of N-GQD-MOF-MIP.

Table S1. The performance of various type of HMF sensors in the literature.

Method	Linear Range	LOD	Reference
HPLC-FLD	0.03 mM–0.21 mM	2.37 μM	[66]
Electrochemical	1.0 pmm to 7 ppm	2.95 μM	[1]
Electrochemical	0.5 ppm–5.0 ppm	1.9 μM	[64]
Electrochemical	0.476 μM–2.22 μM	47.6 nM	[65]
Electrochemical	5.0 μM–1000 μM	4.82 μM	[16]
Fluorescence	0.005 μM–5.0 μM	0.021 μM	[40]
Fluorescence	0.01 μM–5 μM	0.040 μM	[63]
Fluorescence	5.0 nM to 5 μM	4.80 nM	[6]
Fluorescence	5.0 nM to 5.0 μM	30 nM	This work

References

1. Francisco, K.C.A.; Lobato, A.; Tasić, N.; Cardoso, A.A.; Gonçalves, L.M. Determination of 5-hydroxymethylfurfural using an electropolymerized molecularly imprinted polymer in combination with Salle. *Talanta* **2022**, *250*, 123723.
6. Jiang, N.; Li, P.; Sun, S.; Wei, W. A ratiometric fluorescence sensor for 5-hydroxymethylfurfural detection based on strand displacement reaction. *Talanta* **2022**, *238*, 123029.
16. Rebelo, P.; Martins, I.; Pacheco, J.G.; Banegas, R.; Costa-Rama, E.; Moreira, M.M.; Nouws, H.P.A.; Delerue-Matos, C. Molecularly imprinted paper-based electrochemical cell for 5-hydroxymethylfurfural determination in honey. *Microchem. J.* **2024**, *205*, 111410.
40. Sheng, A.; Su, L.; Wang, J.; Xue, T.; Wang, P.; Zhang, J. Hydrazone chemistry mediated toehold strand displacement cascade and its application for 5-hydroxymethylfurfural analysis. *Anal. Chim. Acta* **2020**, *1104*, 110–116.
63. Sheng, A.; Su, L.; Jalalah, M.; Al-Assiri, M.S.; Harraz, F.A.; Zhang, J. Hydrazone chemistry assisted DNAzyme for the analysis of double targets. *ChemComm* **2020**, *56*, 695–698.
64. Khonyoung, S.; Upan, J.; Mool-am-kha, P.; Lerdsri, J.; Jakmunee, J.; Reanpang, P. A rapid and reliable electrochemical determination of 5- hydroxymethylfurfural in honey exploiting nickel oxide nanoparticles modified electrode. *Talanta* **2024**, *268*, 125373.
65. Salhi, I.; Samet, Y.; Trabelsi, M. Direct electrochemical determination of very low levels of 5-hydroxymethyl furfural in natural honey by cyclic and square wave voltammetric techniques. *J. Electroanal. Chem.* **2020**, *873*, 114326.

66. Godoy, C.A.; Valderrama, P.; Furtado, A.C.; Boroski, M. Analysis of HMF and furfural in hydrolyzed lignocellulosic biomass by HPLC-DAD-based method using FDCA as internal standard. *MethodsX* **2022**, *9*, 101774.

Disclaimer/Publisher's Note: The statements, opinions and data contained in all publications are solely those of the individual author(s) and contributor(s) and not of MDPI and/or the editor(s). MDPI and/or the editor(s) disclaim responsibility for any injury to people or property resulting from any ideas, methods, instructions or products referred to in the content.
